# Accuracy of four intraoral scanners in oral implantology: a comparative in vitro study

**DOI:** 10.1186/s12903-017-0383-4

**Published:** 2017-06-02

**Authors:** Mario Imburgia, Silvia Logozzo, Uli Hauschild, Giovanni Veronesi, Carlo Mangano, Francesco Guido Mangano

**Affiliations:** 1 Palermo, Italy; 2 Department of Engineering of the University of Perugia, Perugia, Italy; 3 Department of Research and Development of V-GER, Bologna, Italy; 4 Sanremo, Italy; 5 Department of Medicine and Surgery, University of Insubria, Varese, Italy; 6 Department of Dental Science, University Vita Salute S. Raffaele, Milan, Italy

**Keywords:** Intraoral scanners, Oral implants, Accuracy, Trueness, Precision

## Abstract

**Background:**

Until now, only a few studies have compared the ability of different intraoral scanners (IOS) to capture high-quality impressions in patients with dental implants. Hence, the aim of this study was to compare the trueness and precision of four IOS in a partially edentulous model (PEM) with three implants and in a fully edentulous model (FEM) with six implants.

**Methods:**

Two gypsum models were prepared with respectively three and six implant analogues, and polyether-ether-ketone cylinders screwed on. These models were scanned with a reference scanner (ScanRider®), and with four IOS (CS3600®, Trios3®, Omnicam®, TrueDefinition®); five scans were taken for each model, using each IOS. All IOS datasets were loaded into reverse-engineering software, where they were superimposed on the reference model, to evaluate trueness, and superimposed on each other within groups, to determine precision. A detailed statistical analysis was carried out.

**Results:**

In the PEM, CS3600® had the best trueness (45.8 ± 1.6μm), followed by Trios3® (50.2 ± 2.5μm), Omnicam® (58.8 ± 1.6μm) and TrueDefinition® (61.4 ± 3.0μm). Significant differences were found between CS3600® and Trios3®, CS3600® and Omnicam®, CS3600® and TrueDefinition®, Trios3® and Omnicam®, Trios3® and TrueDefinition®. In the FEM, CS3600® had the best trueness (60.6 ± 11.7μm), followed by Omnicam® (66.4 ± 3.9μm), Trios3® (67.2 ± 6.9μm) and TrueDefinition® (106.4 ± 23.1μm). Significant differences were found between CS3600® and TrueDefinition®, Trios3® and TrueDefinition®, Omnicam® and TrueDefinition®. For all scanners, the trueness values obtained in the PEM were significantly better than those obtained in the FEM. In the PEM, TrueDefinition® had the best precision (19.5 ± 3.1μm), followed by Trios3® (24.5 ± 3.7μm), CS3600® (24.8 ± 4.6μm) and Omnicam® (26.3 ± 1.5μm); no statistically significant differences were found among different IOS. In the FEM, Trios3® had the best precision (31.5 ± 9.8μm), followed by Omnicam® (57.2 ± 9.1μm), CS3600® (65.5 ± 16.7μm) and TrueDefinition® (75.3 ± 43.8μm); no statistically significant differences were found among different IOS. For CS3600®, For CS3600®, Omnicam® and TrueDefinition®, the values obtained in the PEM were significantly better than those obtained in the FEM; no significant differences were found for Trios3®.

**Conclusions:**

Significant differences in trueness were found among different IOS; for each scanner, the trueness was higher in the PEM than in the FEM. Conversely, the IOS did not significantly differ in precision; for CS3600®, Omnicam® and TrueDefinition®, the precision was higher in the PEM than in the FEM. These findings may have important clinical implications.

## Background

Intraoral scanners (IOS) are powerful devices used for optical impressions, and are able to collect information on the shape and size of the dental arches (or the position of dental implants) through the emission of a light beam [1, 2]. In fact, they project a beam or light grid (structured light or laser) onto the tooth surface (or implant scanbodies), and capture, through high-resolution cameras, the distortion that such a beam or grid undergoes when they hit these structures [1, 2]. The information collected by these cameras is processed by powerful software that reconstructs the three dimensional (3D) model of the desired structures [2, 3]. In particular, from the genesis of a "cloud of points" a polygonal mesh is derived, representing the scanned object; the scan is further processed to obtain the final 3D model [2, 3].

The conventional physical detection of impression with trays and materials (alginates, silicones, polyethers) represents a moment of discomfort for the patient [4, 5]; this is particularly the case with sensitive subjects, for example those with a strong gag reflex [6]. In addition, it can be difficult for the clinician, especially in the case of technically complex impressions (for example for the fabrication of long-span implant-supported reconstructions) [5, 7]. The optical impression with IOS solves all these problems: it is well tolerated by the patient, since it does not require the use of conventional materials, and is technically easier for the clinician [4, 8, 9].

The use of an IOS allows the immediate determination of the quality of the impression; virtual 3D models of patients are obtained, which can be saved on computer without physically pouring a plaster model [2, 7, 10]. This saves time and space, and it provides the ability to easily send the models to the laboratory using e-mail, reducing time and costs [2, 7, 9, 10]. The clinician can save money each year on the purchase of impression materials, the fabrication of individual trays, and on casting and shipping of plaster models; it is possible to store virtual models of patients without having to dedicate them a space within the clinic [2, 7, 9, 10]. Not least, the clinician can have a powerful marketing tool for more effective communication with the patient.

To date, IOS are used to obtain study models [11], in prosthesis for the detection of impressions necessary for the modeling and fabrication of a whole series of restorations (single crowns [12], fixed partial dentures [13, 14], and in selected cases, complete fixed arches [15]), but also in the surgical field (integrated in acquisition procedures in guided surgery) [16] and in orthodontics (for the fabrication of aligners and different customized orthodontic devices) [17].

This breadth of applications, together with the undoubted advantages deriving from the use of IOS, have led in recent years to great interest in these machines [2, 3]. Consequently, the industry offers every year new devices, with different features: the widest choice and differences between the various machines can complicate the choice for the clinician [1].

Beyond the operational and clinical differences (speed of use, need of powder, size of the tips) and cost (purchase and management) between different machines, the most important element to be considered should be the quality of the data (mathematics) derived from scanning, defined as “accuracy” [18, 19].

Accuracy is the combination of two elements, both important and complementary: "trueness" and “precision” [18, 19]. The term “trueness” refers to the ability of a measurement to match the actual value of the quantity being measured [19]. An IOS should therefore be able to detect all details of the impression and to generate a virtual 3D model as similar as possible to the initial target, and that little or nothing deviates from reality. In order to detect the trueness of a 3D model derived from intra-oral scanning, it is mandatory to have a reference model with error tending to zero, obtained with industrial machines (coordinate measuring machine - CMM or articulated arms) or with powerful industrial desktop scanners [19]. In fact, only the superimposition of the 3D models obtained with an intraoral device to a reference model (probed with CMM or scanned with powerful desktop machine), through the use of specific software, allows us to evaluate the actual trueness of an IOS [19–21]. Although trueness is the key element for an IOS, it is not sufficient, as it must be accompanied by precision. Precision is defined as the ability of a measurement to be consistently repeated: in other words, the ability of the scanner to ensure repeatable outcomes, when employed in different measurements of the same object [14, 15, 19, 20]. The constant repeatability of the result is of great importance: different measurements of the same object must necessarily be comparable, and differ from each other as little as possible. To measure the precision of an IOS, no reference models are needed: it is sufficient to superimpose different intraoral scans between them, and evaluate to what extent they deviate, using dedicated software [14, 15, 19].

Unfortunately, very few studies in the literature have evaluated the accuracy of the different IOS available on the market [19, 20, 22–26]. The available studies mostly report on first-generation scanners [20, 23–26], and do not deal with the most powerful and recent devices: scientific literature is not as fast as the industry. Moreover, only a few studies have compared the ability of different IOS to capture high-quality impressions in patients with dental implants [19, 26–28].

Therefore, the aim of the present study was to compare the trueness and precision of four of the most recent and powerful IOS, in two different situations: in a partially edentulous maxilla (PEM) with three implants and in a fully edentulous maxilla (FEM) with six implants.

## Methods

### The models

**Fig. 1:**
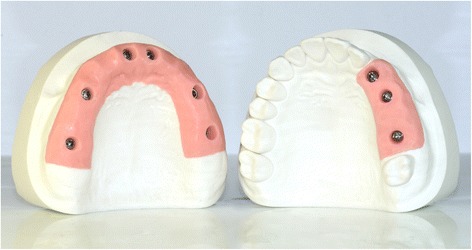
Two different gypsum models were prepared: a partially edentulous maxilla, with three implant analogues in positions #23, #24 and #26, and a fully edentulous maxilla, with the same implant analogues in positions #11, #14, #16, #21, #24 and #26

Two different gypsum models were prepared, representing two different clinical situations. The first gypsum model was a partially edentulous maxilla (PEM), with three implant analogues (BT Safe Int®, BTK-Biotec Implants, Povolaro di Dueville, Vicenza, Italy) in positions #23, #24 and #26; the second gypsum model was a fully edentulous maxilla (FEM), with the same implant analogues in positions #11, #14, #16, #21, #24 and #26 (Fig. 1). After that, nine high-precision non reflective polyether-ether-ketone (PEEK) scanbodies (BT Scanbodies®, BTK-Biotec Implants, Povolaro di Dueville, Vicenza, Italy) were selected. This material was chosen for its optical properties, because it does not reflect light [29]: it is, in fact, well known that IOS may have difficulties scanning reflective, shiny surfaces [3]. These high-precision PEEK cylinders were screwed on the implant analogues, and the models were ready for the evaluation.

### Study design

**Fig. 2:**
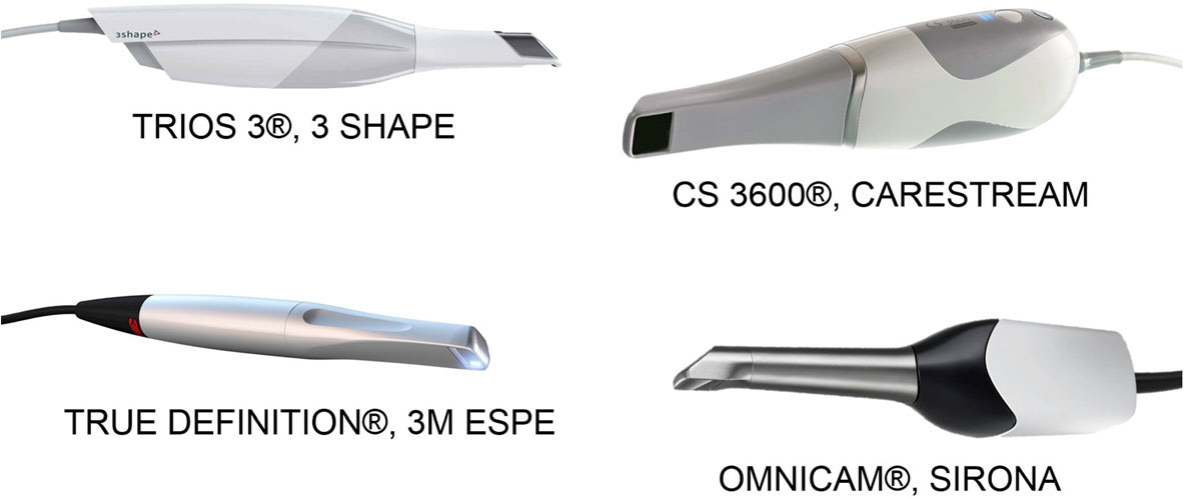
Four different IOS (CS 3600®, Carestream, Rochester, NY, USA; Trios 3®, 3-Shape, Copenhagen, Denmark; Cerec Omnicam®, Sirona Dental System GmbH, Bensheim, Germany; True Definition®, 3M Espe, S. Paul, MN, USA) were compared in this study, with the purpose to investigate their trueness and precision in oral implantology

Four different IOS (CS 3600®, Carestream, Rochester, NY, USA; Trios 3®, 3-Shape, Copenhagen, Denmark; Cerec Omnicam®, Sirona Dental System GmbH, Bensheim, Germany; True Definition®, 3M Espe, S. Paul, MN, USA) were compared in this study (Fig. 2), with the purpose to investigate their trueness and precision in oral implantology. The reference scanner for trueness measurements was an industrial optical desktop scanner (ScanRider®, V-GER srl, Bologna, Italy). The study design was as follows: first, the gypsum models (PEM and FEM) were scanned with the reference scanner, and three scans were taken for each model. All generated datasets were imported into powerful reverse-engineering software (Geomagic Studio 2012®, Geomagic, Morrisville, NC, USA) and superimposed on each other, in order to select one reference dataset (reference model, R1) for the PEM and FEM. The R1 models were then used as references for the trueness measurements of all IOS. In brief, the two gypsum models were scanned with the four IOS. Five scans were then taken for each model, using each different device. The scanning sequence was randomized, in order to reduce the potential negative effects of operator fatigue; the scans were taken sequentially, with an interval of 10 minutes, in order to allow the operator to rest and the device to cool down. A zig-zag scanning technique was followed in all cases, and for each intraoral scanner: starting from the first quadrant (superior right), the tip of the scanner draws an arc movement, from vestibular to palatal and back, slowly moving forward so that teeth, scanbodies and gingiva were scanned from vestibular to palatal (and back), passing over the occlusal plane. In the present study, all IOS were used under the same conditions (in the same room, with a temperature of 20°, humidity of 45%, and air pressure of 760 ± 5 mmHg) by the same dentist with long experience in digital dentistry and intraoral scans.

### The scanners

All information about the reference scanner and the four IOS used in the present study are provided here; the main features of the four IOS are also summarized in Table 1.

**Table 1:** The four IOS used in this study

	Technology of acquisition	Powder	Colour	System
CS 3600®	Active speed 3D video	No	Yes	Completely open – proprietary files (.CSZ) but also open formats (.PLY,.STL) are immediately available
Trios 3®	Confocal microscopy and ultrafast optical scanning	No	Yes	Closed – only proprietary files (.DCM) are available
Cerec Omnicam®	Optical triangulation and confocal microscopy	No	Yes	Closed – proprietary files (.CS3,.SDT,.CDT,.IDT) are available, but with the possibility to obtain open formats (.STL) with Cerec Connect®
True Definition®	Active wavefront sampling 3D video technology	Yes	No	Closed – proprietary files are available, but with the possibility to obtain open formats (.STL) with 3M Connection Center®

### ScanRider® (V-GER srl, Bologna, Italy)

The reference scanner used in the present study was an industrial optical desktop scanner, working under the principle of structured light active triangulation. The device was configurable and composed of four parts: the optical assembly, the 1 or 2° of freedom mechanics, the electronics and the software. ScanRider® features a DLP 600il projector, B/W 1.3 Megapixel cameras and a working distance of 120 mm. It has a standard resolution of 25–50 μm, an average error (accuracy) of 5–10 μm, a precision (standard deviation) of 15–30 μm, a number of triangles for each scan up to 2,500,000 and a free output format (. STL).

### CS 3600® (Carestream, Rochester, NY, USA)

CS 3600® is the second IOS produced by Carestream. It was launched in 2016, and improved based on feedback from the first one, CS 3500® (which was available on the market since 2014). These two IOS differ significantly in the technology of acquisition because CS 3500® used the principle of optical triangulation and generated individual images, while CS 3600® works according to the principle of the active speed 3D video. Both these scanners are available in a USB version, in which the device has a direct connection with the laptop via USB cable; however, the integration of the scanner into the treatment unit has been planned. CS 3600® is a powerful structured LED light scanner; it does not require powder and is able to provide high-quality color images. Such images are a valuable aid in identifying the margin line, when scanning natural teeth. The scanner comes with different sized tips for scanning the frontal and posterior areas. CS 3600® is extremely fast as it allows quick scanning of both jaws, the software acquisition is powerful (in the present study, we have used the software version 1.2.6, released in 30-05-2016) and features a highly intuitive graphical interface. CS 3600® is an open scanner because its produces proprietary files (. CSZ) but also open files (.PLY, STL) that can be opened from any computer assisted design (CAD) software. The use of proprietary files (.CSZ) allows the maintenance of color information, within a dedicated workflow, which involves modeling with proprietary CAD software (CS Restore®) and the subsequent manufacture of a whole series of simple restorations (inlays, onlays, veneers, single crowns and small bridges) with the dedicated in-house milling machine (CS 3000®). On the other hand, the free files (.PLY,.STL) generated by CS 3600® without paying any fee (either monthly or yearly), can be easily opened with any CAD software on the market and therefore manufactured with any milling machine. Therefore, there are no restrictions on the use of such files by laboratories. Through the conventional laboratory workflow, the data acquired from CS 3600® can be used for the manufacture of more complex restorations, such as structures with multiple elements, also supported by implants, as well as frameworks and bars.

### Trios 3® (3-Shape, Copenhagen, Denmark)

Trios 3® is the third IOS fabricated by 3-Shape, after Trios Standard® (2011), which produced monochrome images, and Trios Colour® (2013). Trios 3® was presented in March 2015 at the International Dental Show (IDS) meeting in Cologne, and then launched on the market from May 2015 in three different versions: a trolley version with a touch-screen, a version incorporated into the dental treatment unit, and a USB version. This latter version allows the clinician to use a laptop, into which the scanner is plugged via a USB port; however, this connection is not direct (it requires several connecting cables) and therefore the scanner is not easily transportable. In the last IDS meeting in March 2017, a new wireless version of TRIOS 3® was presented: in this last release, the IOS will connect via Wi-Fi to a laptop or to the traditional cart, eliminating the need for a connecting cable between the scanner wand and the computer. All the aforementioned versions are available with a straight pen-grip handle or with a pistol-shaped handle (320 x 56 x 16 mm). Trios 3® is a powerful and extremely fast structured light scanner. It works under the principle of confocal microscopy and ultrafast optical scanning; it is powder-free and it produces high-quality in-colour images. The scanner has special features integrated, such as the Real Colour Scan®, HD Photo Function® and Digital Shade Determination®: these are interesting because colour scanning can help to differentiate the natural tooth structure and the gingival tissues, and therefore it may help dentists to identify the margin lines. The acquisition software of Trios 3® (in the present study, the software version 16.4 has been used) has automatic artefact elimination and advanced cutting functions, combined with smart blocking functions available for surfaces: the latter feature is very useful when scanning natural teeth, to lock the dental margins highlighted immediately after removal of the retraction cord, and thus avoid overwriting of it. Trios 3® has a big wand, but this is not a limitation because this tip can be used to avoid scanning of unwanted tissues (tongue, cheeks, lips). Like the previous versions, Trios 3® produces proprietary files (.DCM) which can be opened only by the 3-Shape computer-assisted-design (CAD) software (3-Shape Dental System®), via the proprietary cloud-based platform (Trios Inbox®) or setting up a direct connection via Direct Connect®, through which data are fed into the dental system and read out from there. The 3-Shape Dental System® CAD software is extremely powerful and widespread in dental laboratories worldwide. In any case, the scanner does not automatically export files in open formats (.STL,.PLY) readable from other common CAD software: Trios 3® is a closed system; in the present study, therefore, all.DCM files were converted into.STL files using the CAD software Dentalsystem 2016 (version 1.6.3). The CAD software of 3-Shape allows design of all kinds of prosthetic restorations and frameworks (inlays, onlays, veneers, crowns, bridges, bars): in addition, modules for implant (3-Shape Implant Studio®) and orthodontic planning (3-Shape Ortho Analyzer®) are available. However, still 3-Shape has no dedicated milling machines for in-office, chairside restorations.

### Cerec Omnicam® (Sirona, Bensheim, Germany)

Cerec Omnicam® is the last and more powerful of Sirona IOS and it represents the technological evolution of the previous devices (Cerec Bluecam®, available since 2009, and Apollo DI®). Cerec Omnicam® was introduced onto the market in 2012 and is currently available in two different versions: a trolley (Cerec Omnicam® AC) and a tabletop version (Cerec Omnicam® AF). It is a structured light scanner that uses a white LED and it works under the principle of optical triangulation and confocal microscopy. Cerec Omnicam® is fast, it does not require powder and it offers true-colour information. The dimensions of the scanner (228 x 16 x 16 mm) are limited and the tip is not too big, therefore it is easier to scan the posterior areas (maxillary or mandibular third molars). The acquisition software is powerful and it will be further improved with a series of new tools in the last release presented at the recent IDS meeting in Cologne (2017). With Cerec Omnicam®, the digital workflow can take place directly at the chairside, using the proprietary CAD software, or via the cloud-based platform (Cerec Connect®). In fact, Cerec Omnicam® is a closed system, exporting the digital impression data as proprietary files (.CS3,.SDT,.CDT,.IDT) that work only on Sirona’s supporting CAD software and CAM devices. Recently, however, the system has been partially opened, and with Connect®, there is the possibility to transform the proprietary files into.STL files, usable from any CAD system. In the present study, in fact, the software Cerec Connect 4.4.4 has been used, and all proprietary files have been converted into.STL using the Inlab software (16.0). With Sirona, the chairside workflow with the newly launched Chairside software 4.4® and the 3 + 1 axis milling machines Cerec MC® (X/XL) is fully established; the labside workflow includes the inLAB15® CAD software and the MC X5® milling unit. The CAD/CAM system of Sirona allows the clinician and the laboratory to design and mill a series of prosthetic restorations and frameworks (inlays, onlays, veneers, crowns, bridges, bars). In addition, Cerec Omnicam® has special scanning software for orthodontic applications (Cerec Ortho®), which allows digital impressions to be submitted to third-party manufacturers, and also dedicated software for guided surgery (Cerec Guide®), enabling the chairside manufacture of surgical templates for implant placement.

### True Definition® (3M Espe, St. Paul, MN, USA)

True Definition® is the second IOS fabricated by 3M Espe, as it represents the evolution of the LAVA COS® (which was introduced onto the market in 2008), with data processing algorithms that have been altered in order to allow for faster and smoother scanning. True Definition® has been available in the market since 2012, originally only as a trolley version with a touch-screen; however, more recently a new portable version has been introduced with the scanner operating solely on a tablet (True Definition® Mobile). True Definition® is a structured light scanner which uses a pulsating visible blue light, and it works under the principle of active wavefront sampling, a 3D video technology. This scanner requires “dusting” of the surface to be scanned, with titanium oxide powders. These titanium dioxide particles work as randomly distributed landmarks for the optical system. True Definition® produces monochrome images, displayed as a video sequence. True Definition® is not as fast as the other devices used in the present study, but the tip is definitely smaller (the dimensions of the whole scanner are 254 x 16 x 14 mm): this may represent an advantage for the intraoral scanning of posterior regions. Into the acquisition software, the detailed depiction for the analysis of the preparation margin can be performed in 3D; the scanner does not feature a snipping tool, but a rewind function makes it possible to return step-by-step to a desired scan status. In the present study, the software version 5.1.1 has been used. True Definition is a semi-closed system, because data generated during the acquisition must be transferred as proprietary files via a cloud-based platform (3M Connection Center®), but after that there is also the possibility to transform these files into.STL format, upon payment of a monthly fee; in the present study, all files were converted into free.STL. This means that the files can be imported into different CAD software without any limitation. The labside workflow is already established, with the possibility to design all prosthetic restorations (inlays, onlays, veneers, crowns, bridges); in addition, data acquired with True Definition® can be used for planning guided surgery (implant workflow) or orthodontic treatments (orthodontic appliances, clear aligners).

#### Trueness and precision

The calculation of trueness and precision of the digitally acquired 3D models was as previously reported [19]. In brief, all the aforementioned 3D models (the reference R1 models acquired with the powerful desktop scanner, as well as all.STL files obtained with the four different IOS) were imported into powerful reverse-engineering software (Geomagic Studio 2012®, Geomagic, Morrisville, NC, USA). First, the “mesh doctor” function was activated, in order to remove any possible small artefacts or independent polygons present in the models; then, all models were cut and trimmed in order to remove the unnecessary information, using the “cut with planes” function. In order to cut and trim the models in the most uniform possible way, specially designed preformed templates were adopted. The trimmed models were therefore saved into specific folders, and were ready for the superimposition. The superimposition method was first validated and tested through the following procedure, repeated for both the PEM and the FEM. In brief, the reference R1 model was imported into the reverse-engineering software, it was duplicated and moved to another spatial location; these two identical models were then superimposed and registered, and the software calculated the difference between the two surfaces. These tests were repeated five times for each model, and they certified the reliability of the superimposition procedure. After these validation tests, it was possible to proceed with the evaluation of trueness and precision of the four IOS, which proceeded as previously reported [19]. For the evaluation of trueness, the five different 3D surface models obtained from each IOS were superimposed to the corresponding reference model (R1), obtained with the industrial desktop scanner. The superimposition consisted of two different procedures. First, the “three-point registration” function was used: the three points were easily identified on the surface of the implant scanbodies. This function allowed a first, rough alignment of the two 3D surface models to be obtained; after that, the “best fit” alignment function was activated, for the final superimposition and registration. With this function, after defining the reference dataset (R1), as well the parameters for registration (a minimum of 100 iterations were requested in all cases), the corresponding polygons of the selected models were automatically superimposed. An “robust-iterative-closest-point” (RICP) algorithm was used for this final registration, and the distances between the reference R1 and the superimposed models were minimized using a point-to-plane method; congruence between specific corresponding structures was calculated. With this method, the mean (SD) of the distances between the two superimposed models was calculated by the software. A similar procedure was followed for the evaluation of precision of the four different IOS. In this case, however, the reference for superimposition was not the model obtained with the industrial optical desktop scanner (R1), but the 3D surface model obtained from intraoral scanning that, for each of the four IOS, had obtained the best trueness result. Basically in this way, all intraoral scans made with the same scanner were superimposed to this selected 3D surface model; the precision of each IOS could be easily obtained, and again expressed as a mean (SD). Finally, for both the trueness and precision, for an optimal 3D visualization of the results, the distances between corresponding areas of references and all superimposed models were colour-coded, using the “3D deviation” function. A colour map was generated, where the distances between specific points of interest were quantified, overall, and in all planes of space. The colour maps indicated in-ward (blue) or out-ward (red) displacement between overlaid structures, whereas a minimal change was indicated by a green colour. Specific parameters were setting for the different models: for the PEM, the colour scale ranged from a maximum deviation of +200 and −200 μm microns, with the best result given by deviations comprised between +20 and −20 μm (green colour); for the FEM, the colour scale ranged from a maximum deviation of +400 and −400 μm microns, with the best result given by deviations comprised between +40 and −40 μm (green colour).

#### Statistical analysis

A careful statistical analysis was performed, for mean and absolute deviations. Trueness was defined from the comparison between each scan (1 to 5 for every scanner) and the reference model (R1), obtained from the powerful desktop scanner. The analysis was first stratified by the model (PEM and FEM). For each scanner, the mean trueness and its standard deviation (SD) were calculated from analysis of variance, and all possible pairwise comparisons between IOS were tested, using the Tukey method for multiple comparisons. In the Tables’ footnotes, we reported the minimum significant mean differences after the Tukey’s correction, as a guidance for data interpretation. Bartlett’s test was used for the assumption of homoscedasticity of variances across groups. The same analyses were replicated for precision, defined as the comparison between scans made with the same IOS. For these analyses, four comparisons for each scanner were available. Finally, we compared mean trueness and precision of any given scanner by model type (PEM and FEM) using separate *t*-tests, with Satterthwaite approximation for the variance. All statistical analyses were conducted using a powerful statistical package (SAS software release 9.4®, SAS Institute, Cary, NC).

## Results

The superimposition method was found reliable, with the validation tests giving a negligible registration error of 0.002 ± 0.004 μm and 0.224 ± 0.071 μm, in the PEM and in the FEM, respectively.

**Fig. 3:**
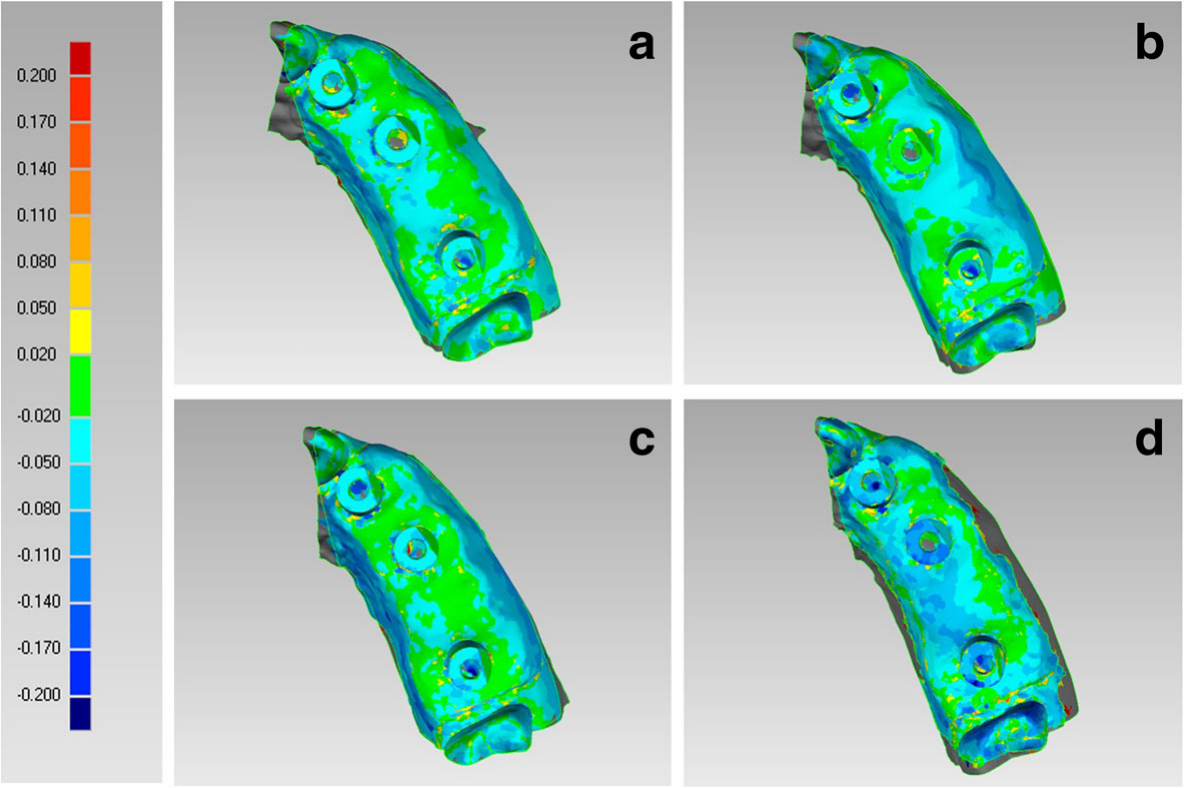
Trueness in the partially edentulous maxilla, occlusal view. The best single result obtained with each device were: (**a**) CS 3600® 44 ± 44 μm; (**b**) Trios 3® 48 ± 52 μm; (**c**) Cerec Omnicam® 57 ± 66 μm; (**d**) True Definition® 57 ± 52 μm

In the PEM, CS 3600® had the best trueness (45.8 ± 1.6 μm), followed by Trios 3® (50.2 ± 2.5 μm), Cerec Omnicam® (58.8 ± 1.6 μm) and True Definition® (61.4 ± 3.0 μm) (Fig. 3). In the PEM, CS 3600® had a statistically higher mean trueness than Trios 3®, Omnicam® and True Definition®; in addition, Trios 3® had a statistically higher mean trueness than Omnicam® and True Definition®. No statistically significant differences were found between Omnicam® and True Definition®.

**Fig. 4:**
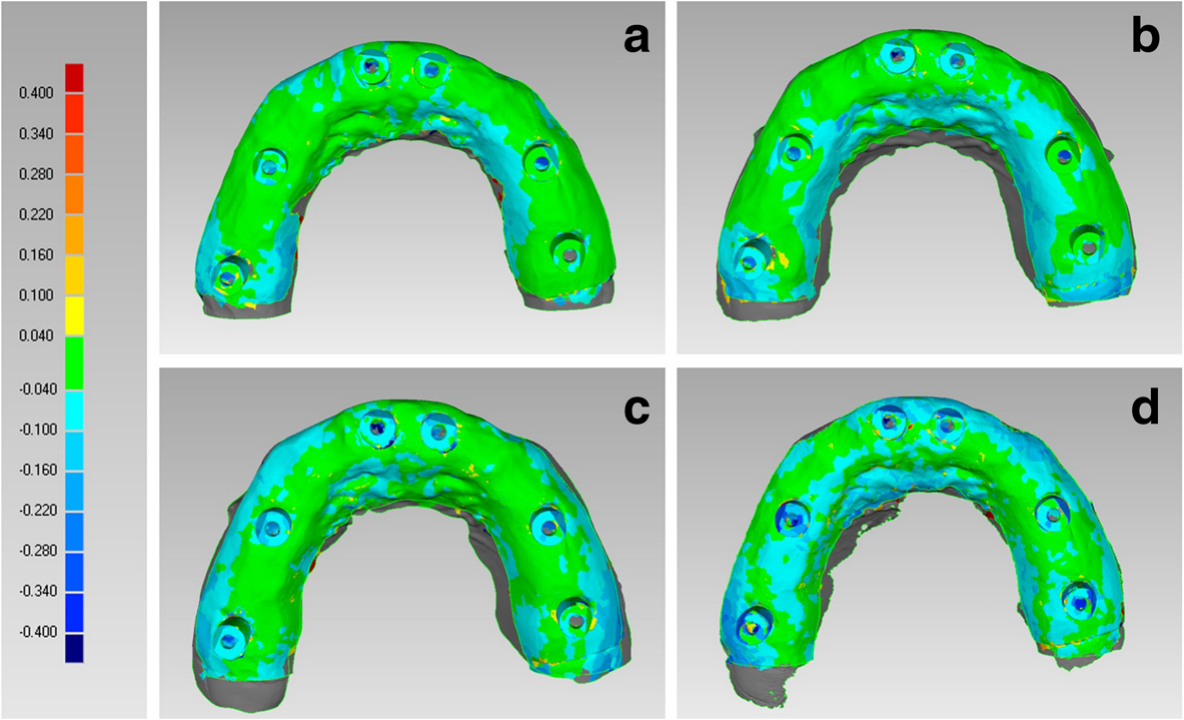
Trueness in the fully edentulous maxilla, occlusal view. The best single result obtained with each device were: (**a**) CS 3600® 50 ± 81 μm; (**b**) Trios 3® 57 ± 89 μm; (**c**) Cerec Omnicam® 63 ± 87 μm; (**d**) True Definition® 84 ± 89 μm

In the FEM, CS 3600® had the best trueness (60.6 ± 11.7 μm), followed by Cerec Omnicam® (66.4 ± 3.9 μm), Trios 3® (67.2 ± 6.9 μm) and True Definition® (106.4 ± 23.1 μm) (Fig. 4). In the FEM, CS 3600®, Trios 3® and Omnicam® had a statistically higher mean trueness than True Definition®. No statistically significant differences were found between CS 3600® and Trios®, CS 3600® and Omnicam®, Trios® and Omnicam®.

Finally, for each of the scanners, the trueness values obtained in the PEM were significantly better than those obtained in the FEM (*t*-test *p*-value <0.05). The trueness values of the four IOS in the PEM and FEM are summarized in Table 2.

**Table 2:** Trueness (mean ± SD), in μm, for partially and fully edentulous maxilla, and *p* values testing the scanner by model interaction

Scanner	Partially edentulous maxilla	Fully edentulous maxilla	*p*-value^1^
	Mean trueness (± SD)	Mean trueness (± SD)	
CS 3600®	45.8 (±1.6) †, ʌ, *	60.6 (±11.7) †	0.047
Trios 3®	50.2 (±2.5) †, ‡, °	67.2 (±6.9) ‡	0.003
Cerec Omnicam®	58.8 (±1.6) ʌ, ‡	66.4 (±3.9) #	0.009
True Definition®	61.4 (±3.0) *, °	106.4 (±23.1) †,‡, #	0.012

*N* = 5 scans for each scanner and model type

The same symbol after SD indicates differences in trueness between scanner pairs (Tukey adjustment for multiple comparison). Minimum signicant difference across scanners 4.1 μm and 24.5 μm for partially and fully edentulous maxilla models, respectively

^1^ *p*-value testing of the interaction between scanner and model type (partially vs fully edentulous maxilla), from t-tests taking into account the heterogeneity of variances (Satterthwaite method). A p-value > 0.05 indicates no difference in scanner trueness according to model type

**Fig. 5:**
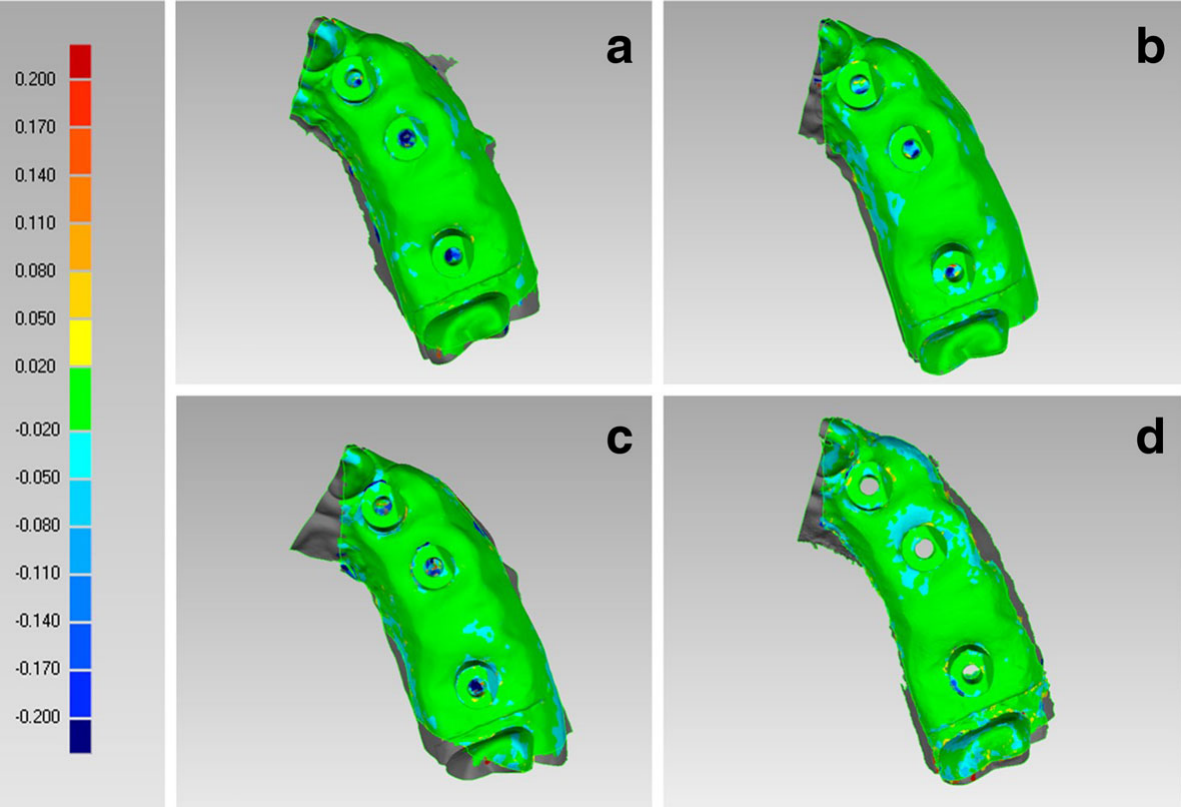
Precision in the partially edentulous maxilla, occlusal view. The best single result obtained with each device were: (**a**) CS 3600® 19 ± 50 μm; (**b**) Trios 3® 21 ± 42 μm; (**c**) Cerec Omnicam® 25 ± 53 μm; (**d**) True Definition® 15 ± 28 μm

In the PEM, True Definition® had the best precision (19.5 ± 3.1 μm), followed by Trios 3® (24.5 ± 3.7 μm), CS 3600® (24.8 ± 4.6 μm) and Cerec Omnicam® (26.3 ± 1.5 μm) (Fig. 5). No statistically significant differences were found between different IOS, in the PEM.

**Fig. 6:**
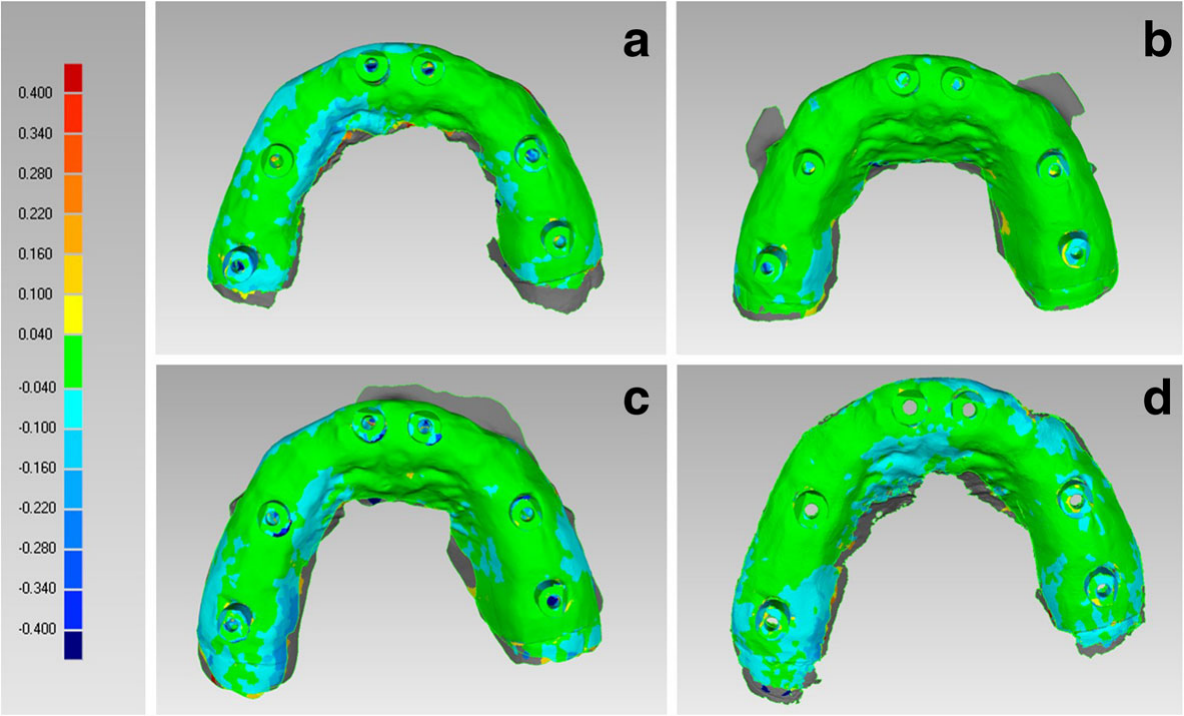
Precision in the fully edentulous maxilla, occlusal view. The best single result obtained with each device were: (**a**) CS 3600® 51 ± 75 μm; (**b**) Trios 3® 24 ± 45 μm; (**c**) Cerec Omnicam® 50 ± 74 μm; (**d**) True Definition® 42 ± 44 μm

In the FEM, Trios 3® had the best precision (31.5 ± 9.8 μm), followed by Cerec Omnicam (57.2 ± 9.1 μm), CS 3600® (65.5 ± 16.7 μm) and True Definition® (75.3 ± 43.8 μm) (Fig. 6). Once again, no statistically significant differences were found among the different IOS, with regard to the precision in the FEM.

For CS 3600®, Omnicam® and True Definition®, the values obtained in the PEM were significantly better than those obtained in the FEM (*t*-test *p*-value <0.05); conversely, for Trios 3®, no statistically significant differences were found in the precision values between the PEM and the FEM. The precision values of the four IOS in the PEM and FEM are summarized in Table 3.

**Table 3:** Precision (mean ± SD), in μm, for partially and fully edentulous maxilla, and *p* values testing the scanner by model interaction

Scanner	Partially edentulous maxilla	Fully edentulous maxilla	*p*-value^1^
	Mean precision (± SD)	Mean precision (± SD)	
CS 3600®	24.8 (±4.6)	65.5 (±16.7)	0.01
Trios 3®	24.5 (±3.7)	31.5 (±9.8)	0.3
Cerec Omnicam®	26.3 (±1.5)	57.2 (±9.1)	0.006
True Definition®	19.5 (±3.1)	75.3 (±43.8)	0.08

*N* = 5 scans for each scanner and model type

The same symbol after SD indicates differences in precision between scanner pairs (Tukey adjustment for multiple comparison). Minimum signicant difference across scanners: 7.2 μm and 51.2 μm for partially and fully edentulous maxilla models, respectively

^1^ *p*-value testing of the interaction between scanner and model type (partially vs fully edentulous maxilla) from t-tests taking into account the heterogeneity of variances (Satterthwaite method). A *p*-value > 0.05 indicates no difference in scanner precision according to model type

As a statistical limitation, we should note that the standard deviation for trueness and precision for the True Definition® instrument was larger than for the remaining IOS, leading to a rejection of the homogeneity of variances for the FEM. However, this was due to one scan with increased trueness, and one with increased precision, with respect to the True Definition® average parameters. When we repeated the analyses after having excluded these observations, we confirmed the findings both for the trueness (differences across scanners) and for the precision (no differences across scanners) parameters.

## Discussion

The digital revolution is radically changing the dental profession, through the introduction of a whole range of devices, software and machines [30, 31]. Today, we can easily switch from real to virtual, through the use of powerful image acquisition systems (intraoral [2, 3], desktop [32] and face scanners [33], and cone beam computed tomography [34]). Within the virtual world, it is possible to plan in detail a whole range of surgical, prosthetic and orthodontic therapies, with the aid of 3D modeling and processing software (software for guided implant surgery, and prosthetic CAD software) [35]. Finally, using new aesthetic materials [36] and powerful machines (such as milling units and 3D printers), we can fabricate surgical guides [16], prosthetic restorations [2, 9, 10, 12–15, 20], and orthodontic appliances [17].

In particular, the IOS are rapidly spreading within the dental clinics, because their use entails significant advantages for the clinician [2]. In fact, IOS allows for the taking of optical impressions of teeth and implants, using only a beam of light. The optical impressions are more comfortable for the patient [4, 5, 7] and easier to take for the clinician [7–10, 12, 14]: therefore, they are rapidly supplanting conventional impressions (with trays and materials), with the latter likely to disappear in the next few years [1, 30].

Several studies and literature reviews have demonstrated that IOS can be a reliable tool for taking impressions of single and multiple abutments in dentate patients [12–15, 37]. However, only a few studies have dealt with the use of IOS in oral implantology [7, 9, 10, 27, 28], and still there is no sufficient evidence on the possibility of using IOS to take impressions for long-span restorations [38–40], or in the case of fully edentulous patients [25, 39].

In addition, little is known about the quality of the different IOS currently available on the market. Only a few studies have compared the trueness and precision of different IOS [20, 22–24, 26–28] and most of these focused their attention on fully dentate models [20, 22–24], whereas scientific evidence on the performance of different devices in oral implantology is rather weak [19, 26–28].

The first study that compared the accuracy of three different IOS in oral implantology was performed by Van der Meer et al. [26]. In this in vitro study, the authors fabricated a stone cast master model, with three implant analogues with high precision PEEK cylinders screwed on; this model was scanned with a powerful industrial optical scanner, in order to obtain a platform for reference measurements, and then with three different IOS (Cerec Bluecam®, Itero® and Lava COS®) [26]. The intraoral scans were then imported into software for superimposition of 3D surface models, and the distances and angulation between the cylinders were assessed, comparing these values with those obtained on the reference model [26]. At the end of the study, the distance errors were the smallest and most consistent for the Lava COS®, whereas they were the largest and least consistent for the Cerec Bluecam®; all the angulation errors, however, were small [26]. The authors concluded that an increase in distance and angular errors should be expected with IOS over the length of the arch, due to the accumulation of registration errors encountered during the progress of the scan in the space [26].

In another study, Ajioka et al. [27] evaluated the accuracy of optical impressions in oral implantology, comparing a virtual model reproduced by an intraoral scanner (Lava COS®) to a working cast made by a conventional silicone impression technique. The authors placed two implants on a master model, and then fabricated plaster casts from the master model by conventional silicone impression [27]. A CMM was used to measure the distances and angulations between the implant abutments, on the master model and on the working casts; the.STL models derived from these measures were then superimposed with the files derived from the Lava COS®, with the aim to evaluate the trueness and precision of the scanner [27]. At the end of the study, the mean trueness of the Lava COS® and working casts were 64.5 μm and 22.5 μm, respectively; the mean of precision of the Lava COS® and working casts were 15.6 μm and 13.5 μm, respectively [27]. The authors concluded that distance errors of the optical impression were slightly greater than those of the conventional impression [27].

In a more recent in vitro study, Mangano et al. [19] compared the trueness and precision of four different IOS in oral implantology, in two different situations: in a partially model (PEM) with three implant analogues and in a fully edentulous model (FEM) with six implant analogues, respectively. Once again, these models were digitized with a powerful optical scanner, used as a reference, and with four IOS (Trios 2®, Carestream CS 3500®, Zfx Intrascan® and Planmeca Planscan®); five scans were taken for each model, using each different IOS [19]. All datasets were loaded into reverse-engineering software, where intraoral scans were superimposed on the reference model, to evaluate general trueness, and superimposed on each other within groups, to evaluate general precision [19]. Moreover, the distances and the angles between simulated implants were measured in each group, and compared to those of the reference model, to evaluate local trueness [19]. At the end of the study, CS 3500® had the best performance in terms of general/local trueness and precision, followed by Trios2® and Zfx Intrascan®; the worst results were reported with Planmeca Planscan®. In detail, CS 3500® had high trueness (47.8 μm) and precision (40.8 μm) in the PEM, but also excellent trueness (63.2 μm) and precision (55.2 μm) in the FEM [19]. Trios2® performed well too, with a trueness and precision of 71.2 μm and 51.0 μm in the PEM, and a trueness and precision of 71.6 μm and 67.0 μm in the FEM [19]. Zfx Intrascan had a trueness and a precision of 117.0 μm and 126.2 μm in the PEM, and a trueness and precision of 103.0 μm and 112.4 μm in the FEM. Finally, Planscan had a trueness and a precision of 233.4 μm and 219.8 μm in the PEM, and a trueness and precision of 253.4 μm and 204.2 μm in the FEM. In this study, significant differences were found between the different IOS [19]. In the PEM, with regard to trueness, Trios® was significantly better than Planscan®, CS 3500® was significantly better than Zfx Intrascan® and Planscan®, and Zfx Intrascan® was significantly better than Planscan®; with regard to precision, Trios® was significantly better than Zfx Intrascan® and Planscan®, CS 3500® was significantly better than Zfx Intrascan® and Planscan®, and Zfx Intrascan® was significantly better than Planscan® [19]. In the FEM, in terms of trueness, Trios® was significantly better than Planscan®, CS 3500® was significantly better than Zfx Intrascan® and Planscan®, and Zfx Intrascan® was significantly better than Planscan®; on the other hand, with regard to precision, Trios® was significantly better than Zfx Intrascan® and Planscan®, CS 3500® was significantly better than Zfx Intrascan® and Planscan®, and Zfx Intrascan® was significantly better than Planscan®.

It is important to note that in the aforementioned work, the authors found no differences in trueness and precision between PEM and FEM [19]; however, this result may be due to the fact that the 3D surface models of the partially edentulous patient were not cut and trimmed, and the related calculations were consequently performed on the whole arch. This can be considered a limitation of this study [19]. In fact, the currently available literature on dentate patients has clearly evidenced how the scanning of single teeth and/or quadrants/sextants is more accurate than that of complete arches [14, 15, 37]. In fact, still the latter procedure seems to have issues, probably due to an accumulation of registration errors of the patched 3D surfaces; consequently, the fabrication of complete fixed full arches remains a challenge, when data are directly acquired with IOS [15, 37].

Moreover, all the aforementioned studies comparing different IOS in oral implantology were performed on first-generation devices [19]; the significant technological advances made in the last months have allowed manufacturers to launch a series of new, extremely powerful devices, in order to make possible the intraoral scanning of full arches in dentate and fully edentulous (implant) patients.

In our present in vitro study, two gypsum models have been prepared with respectively three and six implant analogues and PEEK cylinders screwed on. These models were scanned with a powerful industrial optical desktop scanner (ScanRider®), used as reference, and with four technologically advanced and latest generation IOS (CS 3600®, Trios 3®, Omnicam®, True Definition®). Five scans were taken for each model, using each IOS. Once again, all IOS dataset were loaded into reverse-engineering software, where they were superimposed on the reference model, to evaluate trueness, and superimposed on each other within groups, to evaluate precision. With regard to trueness, in the PEM, CS 3600® had the best results (45.8 ± 1.6 μm), followed by Trios 3® (50.2 ± 2.5 μm), Omnicam® (58.8 ± 1.6 μm) and True Definition® (61.4 ± 3.0 μm). Excellent results were obtained with all devices, compatible with a successful clinical use of all these IOS in similar clinical applications (i.e. the design and fabrication of short-span, implant-supported bridges composed of 3–4 elements). This is in accordance with the current literature [7, 9, 10, 27, 28]. Moreover in our present work, statistically significant differences were found in this application between different IOS (CS3600® was significantly better than Trios3®, Omnicam® and TrueDefinition®; and Trios3® was significantly better than Omnicam® and TrueDefinition®). Although all IOS performed well in the PEM, the statistically significant differences emerging from our study should be taken into account, because a greater accuracy in the acquisition allows design and mill restorations that best suit and adapt into the clinical setting [2, 8, 14–17, 19]. Therefore, the use of CS 3600® would seem preferable in similar clinical settings, in the light of the greater accuracy of the device. In our present work, in the FEM, CS 3600® had the best trueness (60.6 ± 11.7 μm), followed by Omnicam® (66.4 ± 3.9 μm), Trios 3® (67.2 ± 6.9 μm) and True Definition® (106.4 ± 23.1 μm). In this application, very good results were obtained with the first three scanners, with little differences between them; these scanners were significantly better than True Definition®. In light of these results, data derived from the acquisition with the first three IOS could hypothetically be employed to successfully design and manufacture full arches implant-supported restorations: this is important, because until recently, using first-generation scanners, it was difficult or impossible to achieve such accuracy in similar challenging clinical applications [15, 18–20, 25]. The most important element emerging from this study is, however, another, confirming recent evidence that has emerged from the literature [14, 15, 38–40], namely that for each IOS, the trueness values obtained in the PEM were significantly better than those obtained in the FEM. Finally, with regard to precision, no statistically significant differences were found in our present study, between the four different IOS: neither in the PEM nor in the FEM. Excellent results were obtained in the PEM, with minimal deviations from the reference model. In the PEM, True Definition® had the best precision (19.5 ± 3.1 μm), followed by Trios 3® (24.5 ± 3.7 μm), CS 3600® (24.8 ± 4.6 μm) and Cerec Omnicam® (26.3 ± 1.5 μm). In the FEM, the deviations from the reference model were higher, as expected: Trios 3® had the best precision (31.5 ± 9.8 μm), followed by Cerec Omnicam (57.2 ± 9.1 μm), CS 3600® (65.5 ± 16.7 μm) and True Definition® (75.3 ± 43.8 μm). Accordingly for CS 3600®, Omnicam® and True Definition®, the values obtained in the PEM were significantly better than those obtained in the FEM; it is interesting to note, however, that no statistically significant differences were found in the precision values between the PEM and the FEM for TRIOS 3®. In this sense, Trios 3® seems to guarantee high precision in different clinical settings, and this is a clear advantage of this machine.

Our present study has limits. First of all, it is an in vitro study: therefore, the important results obtained here should be necessarily translated into the clinical setting, and validated in vivo, where there are additional factors that can degrade the quality of a scan (such as saliva, blood, limited mouth opening and movements of the patient) [3, 22, 37]. Second, although latest generation and very powerful, the scanner used here as a reference was an optical desktop scanner. The use of a contact scanner (CMM, articulated arm), that can physically probe the surface of the scanned models could be preferable in terms of accuracy [19–21, 27], although it must be remembered that the physical contact with the probe can somehow damage or modify the models, and that contact scanners are slow and expensive. Third, some limitations may be related to the sample size (n = 5 scans for each IOS), even if this seems to be a convenient sample size taking into account similar studies [14, 15, 19–25]. In conclusion, further studies with larger sample size are needed, to confirm the outcomes emerging from the present work; these studies should compare all the latest generation IOS, to provide even more interesting data to clinicians.

## Conclusions

In the present in vitro study, we have compared the trueness and precision of four latest generation IOS (CS3600®, Trios3®, Omnicam®, TrueDefinition®) in two different situations (in a PEM with three implants and in a FEM with six implants, respectively). Excellent results in terms of trueness and precision were achieved with all IOS, scanning the two different models. However, important findings have emerged from our present work. First, significant differences in trueness were found among different IOS: this may have important clinical implications. Since in digital dentistry modeling and milling depend essentially on the data acquired through the optical impression, the use of the most accurate IOS would seem preferable, in order to improve the quality of fit and marginal adaptation of the implant-supported prosthetic restorations. In our present study, CS 3600® gave the best trueness results, therefore it should be preferable to use it in similar clinical settings. Second, the scanning accuracy was higher in the PEM than in the FEM. This indicates that, despite the considerable progress made by the latest generation IOS, scanning a fully edentulous patient remains more difficult than to scan an area of more limited extent, and consequently the design and milling of full-arch restorations on the basis of these scanning data may still present problems. Third, no statistically significant differences were found in the precision, among the four different IOS; however, Trios 3® performed better in the transition from the partially to the fully edentulous model.

## Abbreviations

3D: Three dimensional
CAD: Computer assisted design
CMM: Coordinate measuring machine
FEM: Fully edentulous model
IDS: International Dental Show
IOS: Intraoral scanners
PEEK: Polyether-ether-ketone
PEM: Partially edentulous model
R1: Reference model
RICP: Robust-iterative-closest-point
SD: Standard deviation
